# Case Report: First case of paternal mosaicism in Snijders Blok–Fisher syndrome

**DOI:** 10.3389/fgene.2026.1892974

**Published:** 2026-07-28

**Authors:** Alex Barragán-Muñoz, Paula Trilla, Aurora Sanchez, Miriam Potrony, Irene Madrigal, Laia Rodriguez-Revenga, Maria Isabel Alvarez-Mora

**Affiliations:** 1 Department of Biochemistry and Molecular Genetics, Hospital Clinic of Barcelona, Barcelona, Spain; 2 Fundació de Recerca Clínic Barcelona-Institut d’Investigacions Biomèdiques August Pi i Sunyer (FCRB-IDIBAPS), Barcelona, Spain; 3 CIBER of Rare Diseases (CIBERER), Instituto de Salud Carlos III, Madrid, Spain

**Keywords:** genetic counseling, paternal mosaicism, POU3F3, RNA testing, Snijders Blok–Fisher syndrome

## Abstract

Snijders Blok–Fisher syndrome (SNIBFIS; OMIM #618604) is a rare autosomal dominant neurodevelopmental disorder caused mainly by *de novo* variants in *POU3F3* gene. Here, we report for the first time, two affected siblings harboring a truncating *POU3F3* variant inherited from their apparently unaffected father. Sanger sequencing across multiple tissues (blood, saliva, urine, and hair root samples) in the father together with RNA testing confirmed gonosomal mosaicism. Clinical re-evaluation of the father evidenced a previously unrecognized cognitive impairment. Conventional methods often fail to detect mosaic variants, highlighting the necessity to integrate integration of high-depth sequencing for precise risk evaluation. This work redefines the genetic architecture of SNIBFIS, emphasizing that comprehensive parental testing are indispensable for precise genetic counseling and family planning. The identification of mosaicism highlights the importance of increased clinical awareness, especially in families with more than one affected individual, and supports the use of comprehensive, high-resolution genetic testing strategies for accurate variant detection and characterization.

## 1 Introduction

Pathogenic heterozygous variants in *POU3F3* are responsible for Snijders Blok–Fisher syndrome (SNIBFIS; OMIM #618604), a rare autosomal dominant neurodevelopmental disorder (NDD) characterized by developmental delay (DD), variable degrees of intellectual disability (ID), speech and language impairment, dysmorphic features, hypotonia, as well as behavioral and psychiatric comorbidities. The core clinical features associated with this recently recognized disorder were initially delineated through the description of a cohort of 19 individuals harboring heterozygous *POU3F3* disruptions (Snijders Blok et al., 2019). Recently, larger phenotypic datasets have been reported recruiting novel patients with *POU3F3* variants through international collaborative efforts, enabling further refinement of the associated clinical spectrum. Additional manifestations described include ophthalmological abnormalities, hearing loss, sleep disturbances, gastrointestinal comorbidities, joint hypermobility, and epilepsy, the latter representing a less common but clinically relevant feature with an expanding phenotypic spectrum (Rossi et al., 2023).

*POU3F3* gene is located at cytoband 2q12.1 and contains two functional domains, POU-Specific (POU-S) and POU-Homeodomain (POU-H), which are essential for the site-specific binding to the different target genes. Since *POU3F3* is an intronless gene, transcripts harboring premature termination codons are expected to be relatively insensitive to nonsense-mediated mRNA decay (NMD) (Maquat, 2004), potentially resulting in the expression of truncated proteins. Functional studies based on luciferase assays demonstrated both loss and gain of function mechanisms underlying the molecular basis of this disorder (Snijders Blok et al., 2019). Recent genotype–phenotype studies have been proposed. Loss-of-function variants have been reported to be enriched among individuals presenting with dysmorphic features and sensory abnormalities, whereas epilepsy has been more frequently associated with gain-of-function variants affecting the POU-S or POU-H domains. Notably, no significant differences in the overall severity of DD or ID have been observed between truncating and non-truncating variants (Rossi et al., 2023).

To date, approximately 30 families have been reported, with the majority of cases resulting from *de novo* variants (Torun et al., 2021; Zhang et al., 2023). To our knowledge, only two truncating variants, p. (Ser24Ter) (Rossi et al., 2023) and p. (Arg451Leufs*185) (Snijders Blok et al., 2019) have been reported as inherited form an affected parent, and no instances of germline mosaicism have been described. Here, we report, for the first time, two affected siblings harboring a truncating *POU3F3* variant inherited from their apparently unaffected father.

## 2 Case description

**FIGURE 1:**
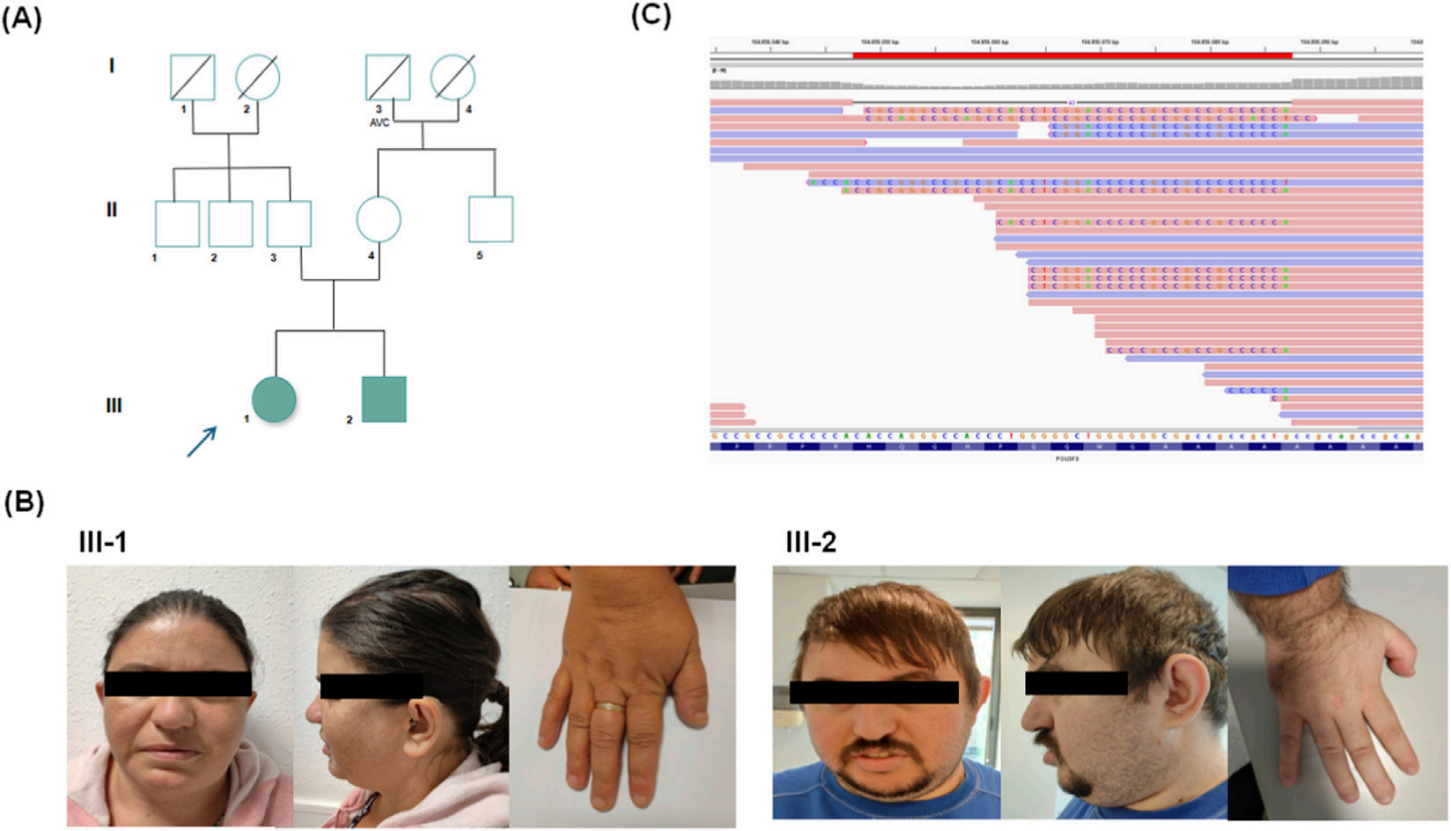
**(A)** Pedigree of the family. Affected siblings are shown as shaded boxes. **(B)** Facial and hand features of: the index case (III-1) and her brother (III-2). **(C)**. View in the Integrative Genome Viewer of the c. 539_578del variant in heterozygosis in *POU3F3* gene.

The family consisted of two affected siblings born from a non-consanguineous Spanish family (Figure 1A). The proband (III-1) was a 38-year-old female referred in 2022 to the Clinical Genetics Unit of Hospital Clínic de Barcelona (Barcelona, Spain) for genetic evaluation due to ID and congenital sensorineural hearing loss.

On clinical examination, the index case (III.1) exhibited global neurodevelopmental impairment with low ID, accompanied by dysmorphic features. These included a narrow, flat forehead, hypotelorism, a high nasal root with a rounded/bulbous tip, a large mouth with downturned corners, a thin upper lip with everted lower lip and chin crease, low-set ears, a broad short neck, hypertrichosis and small hands with short digits, including clubbed/bulbous thumbs and a shortened distal phalanx (Figure 1B).

Family history disclosed an affected brother with more severe ID but without hearing loss. The affected brother (Individual III-2) was a 26-year-old male at the time of clinical evaluation presented with moderate ID, marked obesity and similar dysmorphic features (Figure 1B). Notably, severe deafness was noted as an isolated familial feature in the proband.

Family history was notable for diabetes mellitus in a paternal uncle (II-3) and breast cancer diagnosed at age 48 in the maternal grandmother (II-4), with no prior family history of NDD (Figure 1A).

Written informed consent was obtained from all individuals and their guardians prior to their participation, in accordance with institutional requirements and the Declaration of Helsinki Principles. The institutional review board approved the collection and use of these samples for research purposes (Ethics Committee of Hospital Clinic de Barcelona). In addition, written informed consent for publication of pictures and anonymized clinical and genetic data was further obtained.

## 3 Timeline (Figure 2)

**FIGURE 2:**
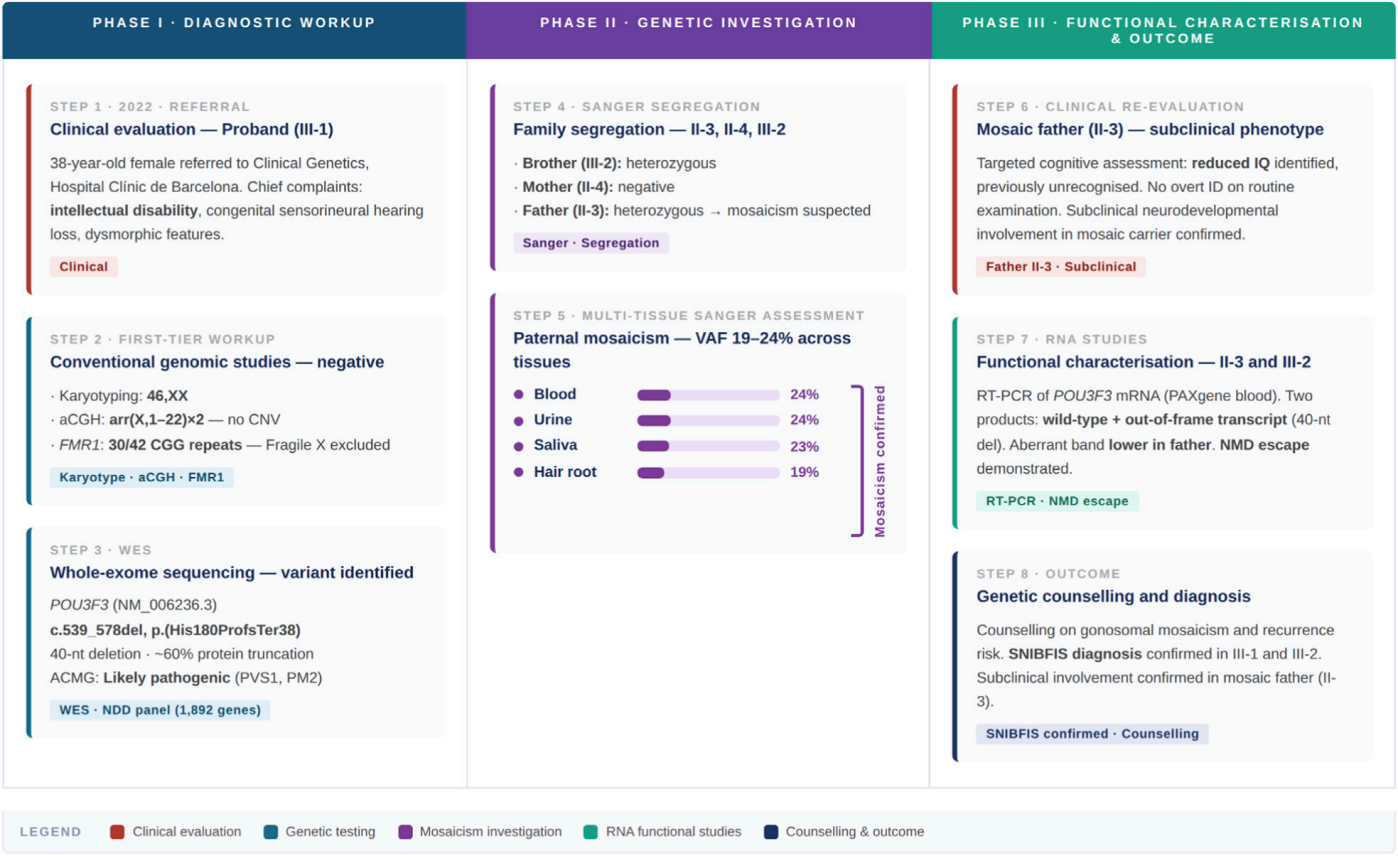
Timeline with relevant data from the episode of care.

## 4 Diagnostic assessment

### 4.1 Genetic studies

DNA extraction was performed from peripheral blood following standard procedures.

In the index case (III-1), the initial genomic studies included conventional karyotyping, chromosomal microarray analysis, and testing for the CGG repeat expansion in *FMR1* gene. Whole exome sequencing (WES) was subsequently performed as previously described (Álvarez-Mora et al., 2022). Briefly, WES libraries were prepared using the DNA Prep with Enrichment (Illumina) and the SureSelect Human All Exon v8 kits (Agilent Technologies, Santa Clara, CA, United States). Paired-end sequencing (2 × 150 bp) of the whole exome was performed with the Illumina NextSeq 2000 sequencer (Illumina, San Diego, CA, United States). Sequence reads were aligned to the human reference genome GRCh37/hg19, and variant calling and annotation were performed using an in-house pipiline (coreBM-Germline_1.0.0).

WES data analysis was based on 1892 genes associated with NDD (available upon request). Potentially pathogenic variants in genes included in the custom panel were filtered according to quality parameters, variant type, pathogenicity predictor scores, and variant frequencies in general population (gnomAD v2). Variant classification was made following the ACMG criteria (Richards et al., 2015).

### 4.2 Segregation analysis

Sanger sequencing was performed to evaluate the presence of *POU3F3* (NM_006236.3) variants in family members (II-3, II-4, III-1, III-2). PCR primers were designed (Forward 5’-ACC CGT CCT CTG TCA AGA TG-3’ and Reverse 5’-TCC GGG CTG CGA GTA GAG-3’) using the Primer3 Input version 4.0.0 web tool (http://primer3.ut.ee/). PCR products were bidirectionally sequenced using BigDye® Terminator version 3.1 Cycle Sequencing Kit (Applied Biosystems, Foster City, CA, United States). Sequencing reactions were run in an ABI Prism 3100XL automated sequencer (Applied Biosystems, Foster City, CA, United States), and the results were analyzed using SEQUENCE® Pilot version 4.0.1 software (JSI medical systemsCorp, New York, NY, United States).

To investigate possible paternal mosaicism, Sanger sequencing was performed on DNA obtained from additional paternal tissue sources, including saliva, urine-derived cells, and hair roots.

### 4.3 RNA functional studies

For individuals II-3 and III-2, 2.5 ml of peripheral venous blood was collected in 5 ml PAXgene tubes (Qiagen, Hilden, Germany). Whole-blood RNA was isolated with the PAXgene Blood RNA Kit (Qiagen) according to the manufacturer’s instructions. RNA quality and quantity were assessed using a Nanodrop ND-1000 spectrophotometer (Thermo Fisher Scientific) and by fluorescence-based quantitation with the Qubit RNA BR assay (Invitrogen).

cDNA was synthesized from 300 ng of total RNA using the High-Capacity cDNA Reverse Transcription Kit according to the manufacturer’s instructions (Applied Biosystems, Foster City, CA, United States). Conventional PCR was performed using the PCR Master Mix (Promega, Madison, Wisconsin, United States) and previously designed custom primers. PCR products were analyzed by 2% agarose gel electrophoresis together with a control sample, and purified using ExoSAP-IT PCR Product Cleanup reagent (Affymetrix, Santa Clara, CA, United States). Purified amplicons were bidirectionally sequenced by Sanger sequencing as described above. Variant effect nomenclature follows the recommendations of the Human Genome Variation Society (den Dunnen et al., 2016).

## 5 Results

Genetic tests performed prior to WES failed to identify the genetic alteration explaining the phenotype in the index case. Analysis of the *FMR1* gene reveled normal alleles (30 and 42 CGGs repeats), excluding Fragile X syndrome. In addition, copy number variants and chromosomal rearrangements were ruled out by chromosomal microarray analysis [arr (X, 1-22)x2] and conventional karyotyping (46, XX).

**FIGURE 3:**
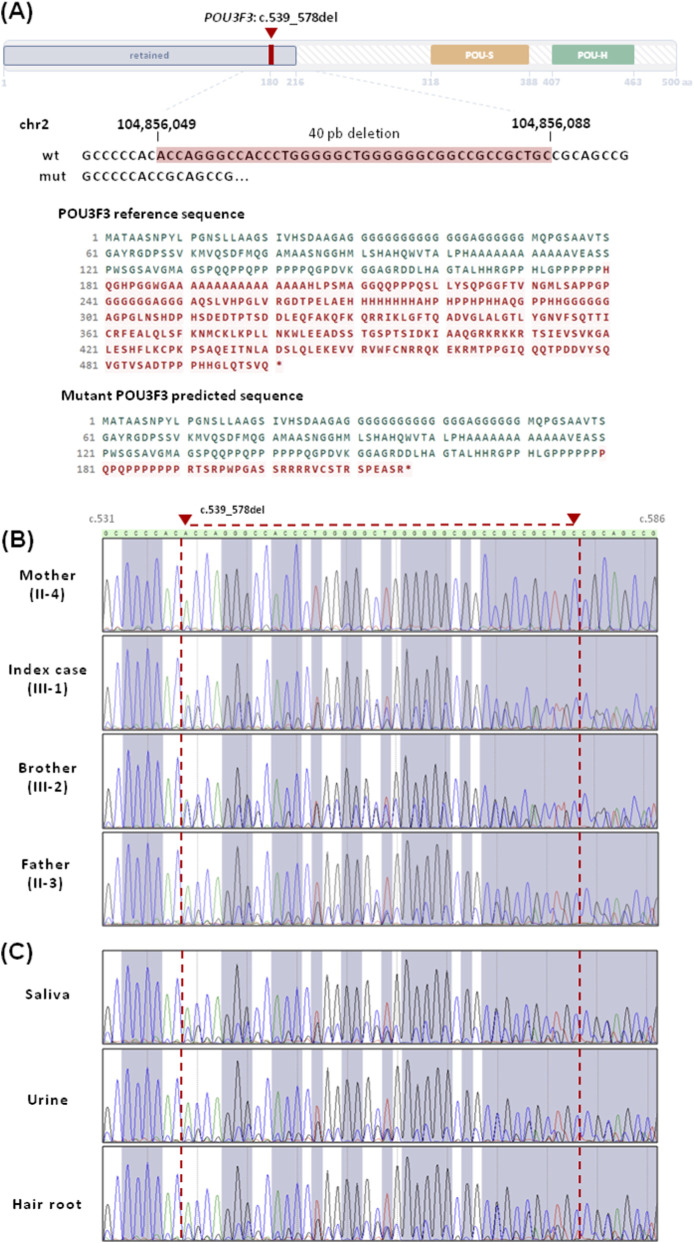
**(A)** Schematic representation of POU domain, class 3, transcription factor 3; **(B)** Segregation analysis of c. 539_578del in blood from all family members; **(C)** Multi-tissue evaluation of paternal mosaicism in: saliva, urine and hair roots cells.

WES identified a heterozygous novel frameshift variant, c.539_578del, p. (His180ProfsTer38) in the *POU3F3* gene (NM_006236.3) (Figure 1C). This variant consists of a 40-nucleotides deletion predicted to truncate approximately 60% of the protein. According to ACMG guidelines, it was classified as likely pathogenic (criteria PVS1 and PM2). Segregation analysis revealed the presence of the variant in the affected brother as well as in her father. Notably, the Sanger sequencing electropherogram form the father showed a reduced signal for the variant, suggesting a possible mosaicism. To confirm this, DNA from additional paternal tissue-urine, hair root, and saliva-was analyzed, revealing an estimated level of ∼24% across all tissues (Figure 3). Subsequent clinical evaluation of the father indicated the absence of overt ID, although cognitive impairment assessment revealed reduced IQ.

**FIGURE 4:**
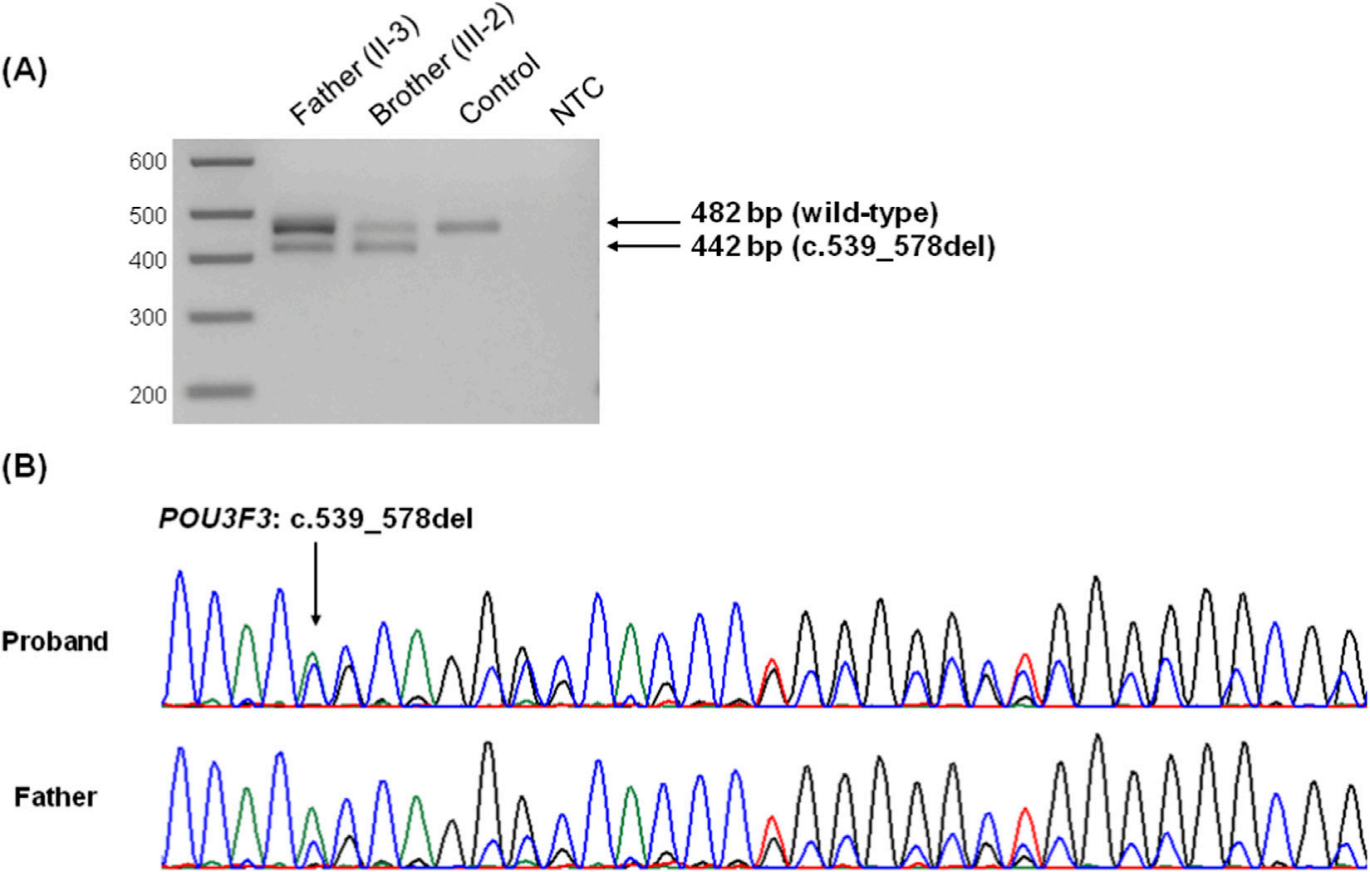
Analysis of *POU3F3* cDNA **(A)** The amplified products of the father (II-3) and the affected brother (III-2) are shown in a 2% agarose gel. **(B)** Sanger sequencing electropherograms of the PCR products.

To further characterize the effect of this variant, RNA analysis was performed in the father and the affected brother. The amplification of *POU3F3* mRNA showed two separate products in both carriers (Figure 4A) Sanger sequencing of PCR products demonstrated the expression of the out-of-frame transcript containing the 40-nucleotides deletion in both individuals. As indicated by the intensity patterns of the bands in the gel as well as the amplification pattern of the RNA sequences electropherograms, our results demonstrated lower expression levels of the aberrant transcript in the father compared with his heterozygous affected son (Figure 4B). Despite the lack of a quantitative method, these results support the presence of the c.539_578del variant in mosaicism.

## 6 Discussion

Although *de novo* variants are generally associated with a low risk of recurrence, a subset of families are at high risk due to parental mosaicism. It is well established that most mutational events occur in the spermatogonial germ cell, turning into an approximately 80% of *de novo* mutation affecting the paternal haplotype (Jónsson H, et al., 2017). The predominance of paternal-origin mutations is likely explained by fundamental differences in gametogenesis. While oocytes remain arrested after meiosis for long periods, spermatogonial stem cells divide continuously throughout life, leading to a greater accumulation of replication errors and DNA damage in the male germline (Waldvogel et al., 2024). Recent studies have estimated the prevalence of parental mosaics in NDD at approximately 3.7% based on trio-WES data (Lecoquierre et al., 2024). This proportion increases to 5%-9% in NDD cohorts enriched for epilepsy phenotypes (Xu X, et al., 2015; Myers CT, et al., 2018), underscoring the clinical relevance of parental mosaicism in apparently sporadic disorders.

Routine clinical laboratory practices for detecting mosaicism remain variable due to differing protocols and the lack of standardized guidelines (Chao et al., 2021). Historically, when a potential disease-causing variant is detected in a proband, Sanger sequencing was used to test parental samples, despite its limitation of detecting mosaicism below 10%. A growing trend advocates switching to NGS-trio methods especially in prenatal diagnosis (Brewer et al., 2020; Chandler et al., 2025). Nevertheless, the VAF sensitivity for detecting parental mosaicism in NGS studies is dependent on both sequencing depth and the bioinformatic pipeline filtering settings. Insights from the PREGCARE research study have emerged Deep-NGS with target depth >5000x, as a reliable strategy to evaluate low-level mosaicism (<1%) (Bernkopf et al., 2023).

Our family broadens the current knowledge of SNIBFIS by providing the first description of paternal mosaicism involving *POU3F3* gene. This syndrome has been predominantly associated with *de novo POU3F3* variants, with only rare instances of vertical transmission previously documented (Snijders Blok et al., 2019; Rossi et al., 2023). In the present family, detection of the variant across multiple paternal somatic tissues >20%, together with recurrence in two affected offspring, strongly supports the occurrence of a postzygotic mutational event in the father involving both somatic and germline cell lineages, consistent with gonosomal mosaicism.

Both affected siblings exhibited phenotypic features concordant with SNIBFIS, including overlapping craniofacial and limb dysmorphisms, while congenital severe hearing loss represented an additional sensory manifestation in the proband. Remarkably, the reevaluation of the apparent *unaffected* father after segregation analysis, revealing subtle neurocognitive involvement detectable only targeted clinical assessment. This observation highlights the potential underrecognition of mildly affected mosaic carriers in clinical practice. In addition, germline mosaicism has serious implications in genetic counseling. The recurrence risk for a presumed *de novo* variant is generally estimated to be 1%-2%, meaning the vast majority of future pregnancies will not be affected. However, in the case of gonadal (restricted to germ cells) or gonosomal (both somatic and germ cells) mosaicism there is a substantial risk of recurrence for further offsprings, reaching up to 50% depending upon the level of parental mosaicism (Bernkopf et al., 2023). Therefore, ascertaining the presence of these types of mosaicism in a mildly or clinically unaffected parent is critical to optimize genetic counseling and inform estimates of recurrence risk.

Unfortunately, as with most genetic conditions, there is no disease-specific treatment for SNIBFIS. Clinical management is therefore supportive and should be tailored to each patient’s individual clinical manifestations and developmental needs. Early intervention programs, including speech and language therapy, occupational therapy, physiotherapy, and specialized educational support, are considered the cornerstone of management for children presenting with NDD. Given the phenotypic variability associated with SNIBFIS, regular multidisciplinary follow-up involving clinical genetics, developmental pediatrics, neurology, psychology or psychiatry, rehabilitation specialists, and other organ-specific specialists is recommended based on individual clinical findings.

The *POU3F3* gene encodes a 500-amino acid POU-domain-containing transcription factor involved in central nervous system development. As a member of the class III POU family, *POU3F3* plays a critical role in cortical neuronal migration, upper-layer neuronal specification, and neurogenesis (Dominguez et al., 2013). To our knowledge, this report represents the first functional evidence of aberrant *POU3F3* transcript expression in a patient with SNIBFIS. Our findings demonstrate that the out-of-frame transcript escapes NMD. Despite the lack of functional validation at protein level, this alteration may results in the absence of protein expression or if synthesized in a nonfunctional protein. We are aware that this is a limitation of the study. Recently, a patient presenting with severe ID, autistic features, and facial dysmorphism was reported to harbor a *de novo* c.-303C>A variant within the 5′untranslated region (UTR) of *POU3F3*, identified through RNA sequencing and DROP analysis (Hayashi et al., 2026). Although this variant is predicted to generate an N-terminal extension comprising 101 additional amino acids, the functional evidence supporting its pathogenicity remains inconclusive.

In summary, potential mosaicism must be thoroughly considered, not only in familial cases but also in all *de novo* mutations assessed by Sanger sequencing approaches. Conventional methods often fail to detect mosaic variants, highlighting the necessity to integrate integration of high-depth sequencing for precise risk evaluation. This work redefines the genetic architecture of SNIBFIS, emphasizing that comprehensive parental testing are indispensable for precise genetic counseling and family planning. The identification of mosaicism highlights the importance of increased clinical awareness, especially in families with more than one affected individual, and supports the use of comprehensive, high-resolution genetic testing strategies for accurate variant detection and characterization.

## 7 Patient perspective

Informed consent was obtained from all subjects involved in the study.

The molecular diagnosis established the genetic basis of the NDD in both affected siblings, providing a definitive explanation for their lifelong clinical manifestations after many years without a diagnosis. Although no disease-modifying treatment is currently available for SNIBFIS, identifying the molecular cause ended the diagnostic odyssey and enabled accurate genetic counseling for the family.

The identification of paternal gonosomal mosaicism prompted a detailed clinical re-evaluation of the father, who had previously been considered unaffected. During this assessment, he retrospectively reported having experienced learning difficulties during childhood and described that he had always struggled academically at school, despite never having received a neurodevelopmental diagnosis. This case illustrates how mildly affected mosaic individuals may remain unrecognized until molecular testing is undertaken following the diagnosis of an affected relative.

Although the psychosocial impact of this late diagnosis was not formally evaluated, identifying the underlying molecular cause provided a biological explanation for his lifelong learning difficulties and substantially refined genetic counseling for the family. More broadly, obtaining a molecular diagnosis can help families better understand the condition, reduce diagnostic uncertainty, and facilitate informed decision-making regarding future reproductive options. The identification of gonosomal mosaicism in this family also highlights the importance of investigating parental mosaicism, particularly when recurrence is observed, as it has direct implications for recurrence risk estimation and family planning.

## Data Availability

The datasets presented in this article are not readily available because of ethical and privacy restrictions. Requests to access the datasets should be directed to the corresponding author/s.
